# Temporal Mobility Networks in Online Gaming

**DOI:** 10.3389/fdata.2019.00021

**Published:** 2019-06-25

**Authors:** Essa Alhazmi, Nazim Choudhury, Sameera Horawalavithana, Adriana Iamnitchi

**Affiliations:** ^1^Computer Science and Engineering, University of South Florida, Tampa, FL, United States; ^2^Computer Science and Information Technology, Jazan University, Jizan, Saudi Arabia

**Keywords:** online games, mobility networks, online social network (OSN) activities, multiplayers online games, mobility diffusion

## Abstract

This data-driven study focuses on characterizing and predicting mobility of players between gaming servers in two popular online games, Team Fortress 2 and Counter Strike: Global Offensive. Understanding these patterns of mobility between gaming servers is important for addressing challenges related to scaling popular online platforms, such as server provisioning, traffic redirection in case of server failure, and game promotion. In this study, we build predictive models for the growth and the pace of player mobility between gaming servers. We show that the most influential factors in predicting the pace and growth of migration are related to the number of in-game interactions. Declared friendship relationships in the online social network, on the other hand, have no effect on predicting mobility patterns.

## 1. Introduction

Online gaming is not only a multi-billion dollar industry (Anderton, 2017) entertaining a large global population, but also a popular form of social interaction among millions of individuals. As online gaming exercises different types of sociability, such as shared activity (Zhuang et al., 2007; Merritt et al., 2013), tie and team formation (Alhazmi et al., 2017), trust formation (Depping et al., 2016), and long-term associations (McEwan et al., 2012; Jia et al., 2015), it becomes a rich source of temporal social interaction data that can be exploited for many computational social science questions. Data from online gaming environments were used to measure otherwise difficult to observe behaviors, such as cheating (Blackburn et al., 2014; Zuo et al., 2016), toxicity (Kwak et al., 2015), gold mining (Ahmad et al., 2009), and measuring online social capital (Molyneux et al., 2015).

Another human behavior that digital records from gaming environments can describe is mobility. Understanding players' mobility between gaming servers is important in multiple aspects, such as server provisioning, traffic redirection in case of server failure, and game promotion. In addition, the migratory patterns of players can be leveraged in modeling information dissemination or behavior adoption. For example, a player may introduce a new set of gimmicks, or may affect the server culture via positive or toxic social behavior.

In real world, human mobility has been shown to be a socially embedded phenomenon (Bilecen et al., 2018), which is affected by both socio-economic factors and the subjectivity of human behaviors (Barbosa Filho et al., 2011). Two important factors have been observed to contribute toward individual's migration decision (Blumenstock and Tan, 2016). Firstly, the extent to which a migrant is connected to communities at home and at the destination, and secondly, the strength and the support of destination ties in providing access to resources available in the destination environment (e.g., job information). The online gaming environment has different characteristics, and it is unclear whether the same arguments apply to player mobility.

This paper quantifies the importance of in-game interactions for a player's decision to migrate from one server to another within the same game. Players move to different servers over time due to various reasons, including technical performance (latency, computation speed), server/game preferences, peer familiarity, or personal endorsements. Previous studies showed that players tend to join games repeatedly with a set of familiar players with whom they shared past experience (Jia et al., 2015; Alhazmi et al., 2017). In this study, we specifically focus on the social interactions as a factor to characterize players' mobility patterns. We develop machine learning-based models to predict, first, the popularity of players over time with respect to the number of neighbors following their mobility patterns, and second, how fast a player moves between servers relative to the others. We present our results using data from two popular online games, Team Fortress 2 (TF2) and Counter Strike: Global Offensive (CSGO), that involve millions of players across a thousand servers over 4 months.

The contributions of this paper are 3-fold: First, it empirically characterizes mobility patterns of players across servers through the temporal mobility networks mechanism built upon their interactions. Second, it identifies the features relevant to the prediction of players' popularity, including early and late movers in the temporal mobility networks. Finally, it shows empirically that the growth and the pace of the mobility can be predicted.

## 2. Dataset

The gaming dataset used in this study was obtained from two sources: GameMe and the Steam Community. GameMe is a statistical reporting service that monitors real time playing activities on a collection of games. It provides APIs to collect real-time statistics of each player's gaming activity over a thousand gaming servers. The Steam Community is an online social network built on the Steam platform. It also provides APIs to extract players' list of friends, owned games, and game statistics for the most recent 48 h.

We focus on two highly popular games on the Steam platform, CSGO and TF2. CSGO is a tactical combat first person shooter video game where players compete as part of the terrorist or the counter-terrorist team. TF2 is a team-based and objective-oriented first-person shooter game, where players compete on two different teams and can pick a role from different categories, such as pyro, medic, scout, or soldier. The games have similar features including a wide variety of weaponry, maps, in-game voice chat, etc.

We collected data on friendship and temporal gaming interactions in these games through a web crawler that uses the APIs provided by Steam and GameMe. In CSGO, the duration of the collected data range from February 16 to August 9, 2017 (175 days), whereas in TF2, it is from February 16 to April 7, 2017 (51 days). The final dataset recorded over 13 million observations of 1.62 million players and 934 servers in CSGO. For TF2, the dataset contains over two million observations of 231 thousands players in 344 servers. BOT accounts and spectators (i.e., inactive players) were removed from the final dataset.

A game server is an authoritative host of game matches. Online multiplayer gaming environments, such as first-person/third-person shooter games, and role-playing games, provide a list of servers hosting active matches for players. Players can select server(s) and game matches based on different criteria, including server name, player count, match mode, and network latency.

**Figure 1:**
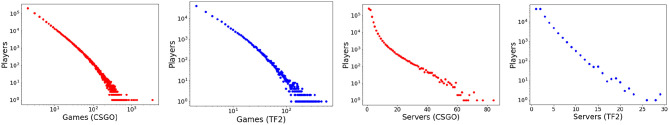
Distribution of players across games and servers in both CSGO and TF2.

Servers in online gaming have variable lifespans. The lifespan of a particular server is the duration of that server being active excluding intermittent downtime. In CSGO, the average server lifespan was 66 days (maximum 102 days) whereas in TF2, it was 39 days (maximum 51 days). Similarly, the average number of matches in CSGO was 1, 245 (maximum 7, 146) in comparison to 228 (maximum 3, 103) found in TF2. Figure 1 shows the distributions of players in matches and servers for both CSGO and TF2.

From this dataset we constructed two social networks for each game: a friendship network based on declared relationships in Steam Community, and an interaction network based on the observed activities at gameme.com. The interaction network temporally connects players in the same match. Thus, an edge in the interaction network is undirected, weighted with the number of observed interactions between the players, and labeled with the list of timestamps when the players were observed in game. Only the active players observed in GameMe are included in the friendship network. Table 1 summarizes the characteristics of interaction and friendship networks in both games.

**Table 1:** Data characteristics of the interaction and friendship networks.

**Game**	**Period**	**Servers**	**Network**	**Players**	**Edges**	**Density**	**NCC**
CSGO	02/16–08/09/2017	934	Interaction	1,106,652	27,415,330	4.48e-05	4,481
			Friendship	928,863	9,525,587	2.21e-05	2,068
TF2	02/16–04/07/2017	344	Interaction	224,922	6,920,096	2.74e-04	1,636
			Friendship	154,038	832,944	7.02e-05	4,258

*NCC, # connected components*.

## 3. Temporal Mobility Networks

In team-based online games, players often follow each other across servers in order to have fun, or to improve their skills and team performance. This study analyzes temporal interaction patterns among players to understand whether co-playing experience has impact on players' movements.

To capture the pattern of players following other players from one server to another, we model players' move as directed networks called temporal mobility networks built on top of the underlying interaction network. Intuitively, players' movements across servers can be explained by social interactions, common experiences related to the characteristics of the home server (e.g., over or under-populated, players' skill, etc.), personal factors (such as the player moving to a different geographical location), and many others. We only capture in this study—due to the inherent limitations of the dataset we collected—the possible reasons due to shared experiences, thus captured by the in-game interactions.

We define a temporal mobility network *G* = (*V, E*) in which nodes are players and a directed link from node *u* to *v* exists if (i) *v* moved to server *S* at time *t*_*m*_; (ii) *u* moved to server *S* at time *t*_*n*_ > *t*_*m*_; and (iii) nodes *u* and *v* have preceding interactions at time *t*_*i*_ < *t*_*m*_. In this context, node *u* is considered to adopt/follow node *v* in his movement to server *S*. We build a temporal mobility network based on the player movements in a given server. Therefore, for a given server, in the corresponding mobility network's context, “mover” and “adopter” will be used interchangeably in the rest of the text. The network is acyclic and only the earliest (first) move to a particular server by a pair of players is considered. The edges are time stamped to allow the study of temporal patterns.

Table 2 presents the main statistics on the mobility networks for both games and servers in games. Servers in the mobility networks are the destinations in the mobility process. Each server will attract disconnected networks of players. The number of disconnected groups (temporal mobility networks) per server for the two games are similar: on average, four groups join each server. The maximum number of mobility networks for two games was 15 and 10, respectively. However, larger groups move in CSGO (maximum is above 8,000 players) compared to TF2 (where maximum is under 3,000 players).

**Table 2:** Basic statistics of mobility networks in each game.

**Games**		**# Nodes per network**				**# Networks per server**		
	**# Networks**	**Min**	**Mean**	**Max**	**# of servers**	**Min**	**Mean**	**Max**
CSGO	2,816	2	202	8,434	705	1	4	15
TF2	1,316	2	51	2,937	323	1	4	10

**Figure 2:**
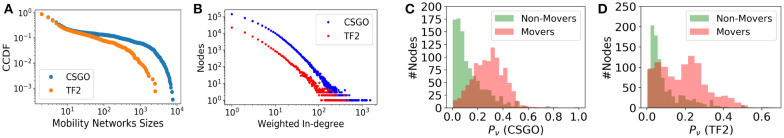
**(A)** CCDF of temporal mobility networks sizes and **(B)** the average weighted in-degree distribution of mobility networks. Distribution of players' neighborhood ratio (*P*_*v*_*i*__) in **(C,D)**.

The distribution of networks' sizes is highly skewed across servers in both games. Figure 2A presents the complementary cumulative distribution functions (CCDF) of the mobility networks' sizes, calculated by considering the total number of nodes per network, and reveals heavy-tailed distributions. Figure 2B shows the average weighted in-degree distribution of players in the mobility networks.

In order to understand what might make players move to a different server, we calculated the ratio *P*_*v*_*i*__ for a player *v*_*i*_ between player's neighbors who moved with respect to all his neighbors as depicted in Easley and Kleinberg (2010). We weigh the number of neighbors by the number of interactions. Figures 2C,D represent sampled distributions over 1,000 movers and non-movers in both games. It appears that the players who do not move have a lower ratio of players who moved in their neighborhoods.

## 4. Prediction Tasks

We have two prediction objectives: (i) identify the popular players in the early stage of the mobility networks formation, and (ii) distinguish early and late movers over the lifetime of the mobility networks. The underlying objectives behind these two classification tasks are complementary. First, the identification of popular players helps us detect whether a particular mobility network grows during our observational period. Second, the classification of early/late movers measures the speed of growth. We also examine the features that are most useful for the two prediction tasks.

### 4.1. Methodology

For the first task, we select temporal mobility networks with lifespans as long as our observation period. We extracted 178 such mobility networks in CSGO and 82 in TF2. We split the network lifespans into four quartiles. We define a node's popularity growth by comparing its in-degree as observed in the first quartile with its in-degree in the last quartile. We consider a node as being popular if its growth is higher than the median of the nodes' growth in that particular mobility network. The classification dataset is constructed by considering each node (player) as a prospective candidate of being popular or non-popular. Each datapoint is described by a set of features (listed in Table 3) constructed from the structural properties of each node in the mobility networks in the earlier stage. These features were used as input to a supervised learning algorithm, Random Forest, to predict the popular nodes in the later phase of the mobility network. The ratio of the training and testing datasets was 3:1 (75% training data, 25% testing data out of 140 thousands and 14 thousands instances in CSGO and TF2, respectively). The two datasets are nearly balanced: 57% in CSGO and 59% in TF2 are nodes in the non-popular category.

**Table 3:** Features used in the pace (P) and growth (G) prediction tasks.

**Features**	**Description**	**Task**
Weight	Weight of edge to the parent node	P
In-degree	Node in-degree	G&P
In-degree_*NF*_	Node in-degree from non-friends.	G
In-degree_*F*_	Node in-degree from friends.	G
Out-degree	Node out-degree	G&P
Out-degree_*NF*_	Node out-degree toward non-friends	G
Out-degree_*F*_	Node out-degree toward friends.	G
Weighted In-degree	Sum of the weighted in-degree.	G
Adoption Rate	Total #adopters per unit time for the node	G
CC_*out*_	CC of out-going edges	P
CC_*in*_	CC of in-coming edges	G&P
CC-NF_*in*_	CC of in-coming edges from non-friends	G
CC-F_*in*_	CC of in-coming edges from friends	G
Time Lag/Adoption Duration	Interval between the first and last adoption	G
In-degree_*parent*_	The in-degree of the node's parent	P
Out-degree_*parent*_	The out-degree of the node's parent	P
CC-parent_*out*_	The parent's CC_*out*_	P
CC-parent_*in*_	The parent's CC_*in*_	P
isFriend	If node and its parent are friends	P

*CC, clustering co-efficient*.

**Figure 3:**
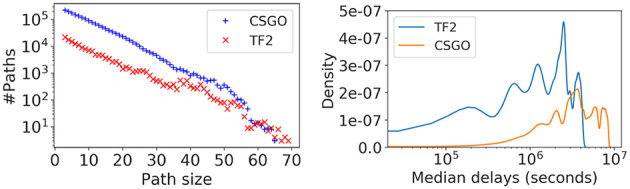
Distribution of path sizes vs. the number of paths and the probability distribution of movement delay by considering temporal paths in mobility networks.

For the second task, predicting the pace of growth, we classify nodes in the mobility networks as early and late movers. We extracted a set of temporal-paths from each mobility network formed in this study using pathpy (Scholtes, 2017). A temporal path consists of a sequence of edges in the network ordered by the node migration time. In Figure 3 (left), we present the distribution of temporal paths by their size. We notice CSGO consists of relatively longer chains of migrations than TF2. (Note that a node may end up joining multiple mobility networks at different times). We discriminate nodes between early and late considering their delay in movement compared to the median delay of the path they belong to: from the list of nodes in each temporal path, nodes having delays shorter than the median value are considered early movers. Figure 3 (right) presents the distribution of median delays from all temporal paths extracted from the largest mobility network in each server of the two games. Interestingly, the sequence of movements observed in TF2 occurs at faster rate than in CSGO.

To predict the pace of gamers' movement, we extracted node-specific features described in Table 3. These features were used as input to the classifier to predict early (class 0) and late (class 1) adopters. We use a Long-Short Term Memory network for the classification task that consists of two blocks of memory-cells with two different layers of hidden units. The first layer contains 32 and the second one contains 8 units. We used the Adam algorithm with 0.001 learning rate as optimizer. We split the temporal-paths set of the mobility networks into two sets: the training set includes 60% of the paths out of 1.7 millions and 155,281 paths in CSGO and TF2 consecutively, while the testing set contains the remaining 40% of paths.

### 4.2. Results

**Figure 4:**
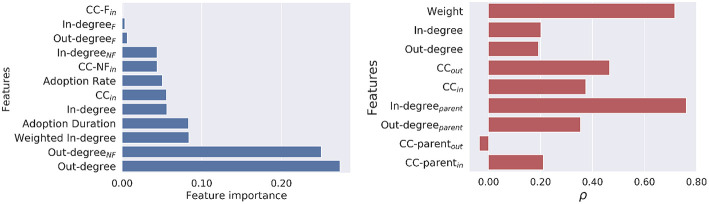
**(Left)** Ranking of features importance in predicting the popularity. **(Right)** Features importance by Spearman's rank correlation coefficient *ρ* between the predicted outcome and the ground-truth in predicting the early/late movers.

For classifying the popular players from unpopular ones, Table 4 shows that Random Forest achieved high recall but low precision. Similarly, the prediction performance in CSGO outperformed the performances in TF2. The underlying reasons behind the better performance are the size of the classification datasets and rich feature values without significant overlap between positive and negatively labeled data points. The list of features are ranked according to their importance, calculated by the Random Forest classifier in CSGO, in Figure 4 (left). It is noteworthy that similar results for TF2 are omitted due to space constraints. The out-degree of a node was found to be the most important feature in predicting the player's popularity. More surprisingly, the out-degree of a node toward his neighbors absent in its neighborhood of the friendship network were found to be most important features in both games. It is evident that friendship has minimal impact in predicting the number of players moving toward a new server following others.

**Table 4:** Prediction results for the popularity in the mobility networks of both games using Random Forest.

**Game**	**Accuracy**	**Class**	**Precision**	**Recall**	**F1-score**
TF2	0.73	1	0.54	0.72	0.61
		0	0.85	0.73	0.79
CSGO	0.75	1	0.62	0.76	0.68
		0	0.85	0.75	0.80

*Class 1 denotes popular nodes and class 0 otherwise*.

For classifying early adopters from late ones, Table 5 presents prediction performances demonstrated by both the Random Forest classifier and the LSTM-based neural network. As intuitively expected, the performance demonstrated by the LSTM has outnumbered the performance by the Random Forest classifier. The underlying reason behind the performance improvement by LSTM is its capability of learning the sequence data and consecutive dependency between feature values to successfully classify binary labels. Improved performance by LSTM also proves that in this context, recurrent neural networks can be a better classifier due to the temporal nature of the mobility network paths. Due to the improved performance by the LSTM over Random Forest classifier, the feature importance of the pace prediction tasks for both games were presented as the Spearman's rank correlation coefficient ρ between the predicted outcomes vs. the ground truth of the test data, as shown in Figure 4 (right). It is noteworthy to mention that similar correlation was observed in TF2. The results demonstrate that the in-degree of a node's parent in the temporal path of the mobility network works as the best performing feature. Alternatively, the weighted interaction between the nodes and their parents with large number of followers are the principal determinants in predicting their pace of movement. On the contrary, the clustering co-efficient of the nodes' parents by considering their out-degree neighbors were found to have negative Spearman correlation in both games. Finally, the friendships between nodes and their parents represent only a small proportion of the instances in both games (2%). Thus, it is irrelevant to measure the correlation of the features incorporating the friendship networks.

**Table 5:** Prediction results for the movement pace in the mobility networks of both games.

**Classifier**	**Game**	**Accuracy**	**Class**	**Precision**	**Recall**	**F1-score**
LSTM	TF2	0.70	1	0.70	0.72	0.71
			0	0.70	0.68	0.69
	CSGO	0.72	1	0.70	0.77	0.73
			0	0.73	0.76	0.70
RF	TF2	0.66	1	0.67	0.67	0.67
			0	0.65	0.65	0.65
	CSGO	0.69	1	0.67	0.76	0.71
			0	0.71	0.62	0.66

*Class 1 denotes late adopters and class 0 otherwise. LSTM denotes Long-Short Term Memory and RF denotes Random Forest*.

## 5. Summary

This study focused on modeling the temporal mobility patterns of online gamers by tracing the chronological movement of players between two servers. We developed two machine learning-based prediction strategies to predict the growth and pace (speed) in the mobility networks. Our main finding is that a player's mobility decision is affected by the co-players with the maximum number of interactions and not by the declared friends in the friendship network. This study can further be extended to explore the impact of community-level network structure over player's mobility across servers.

## Data Availability

The datasets generated for this study are available on request to the corresponding author.

## Author Contributions

The data were collected by EA. The experiments were conceived by AI and conducted by EA and SH. The data were analyzed and interpreted and the manuscript was written by EA, AI, and NC. All authors reviewed the manuscript.

### Conflict of Interest Statement

The authors declare that the research was conducted in the absence of any commercial or financial relationships that could be construed as a potential conflict of interest. The handling editor and reviewer SG declared their involvement as co-editors in the Research Topic, and confirm the absence of any other collaboration.
